# Changes and specificities in health behaviors among healthcare students over an 8-year period

**DOI:** 10.1371/journal.pone.0194188

**Published:** 2018-03-22

**Authors:** M. P. Tavolacci, J. Delay, S. Grigioni, P. Déchelotte, J. Ladner

**Affiliations:** 1 Clinical Investigation Center 1404, IRIB, Rouen University Hospital, Rouen, France; 2 Rouen University Hospital, Inserm U1073, IRIB, Rouen, France; 3 Rouen University Hospital, Department of Nutrition, Rouen, France; 4 Rouen University Hospital, Department of Epidemiology and Health Promotion, Rouen, France; University of California San Diego School of Medicine, UNITED STATES

## Abstract

**Background:**

Healthcare students are future health care providers and serve as role models and coaches to enhance behaviors for healthy lifestyles. However healthcare students face multiple stressors that could lead to adopting risk behaviors.

**Objectives:**

To assess the changes in health risk factors among healthcare students between 2007 and 2015, and to identify specific health behaviors based on the curriculum in a population of healthcare students:

**Methods:**

Two cross sectionnal studies were conducted in 2007 and 2015 among nursing, medical, pharmacy, and physiotherapy students (Rouen, France). During compulsory courses and examination sessions students filled self-administered questionnaires on socio-demographic characteristics and behavior as: tobacco smoking, alcohol consumption, cannabis consumption, eating disorders, regular practice of sport, perceived health, stress and use of psychotropic drugs.

**Results:**

2,605 healthcare students were included (1,326 in 2007 and 1,279 in 2015), comprising 1,225 medical students (47.0%), 738 nursing students (28.3%), 362 pharmacy students (13.9%), and 280 physiotherapy students (10.8%). Between 2007 and 2015, occasional binge drinking and regular practice of sport increased significantly among healthcare students, respectively AOR = 1.48 CI95% (1.20–1.83) and AOR = 1.33 CI95% (1.11–1.60), regular cannabis consumption decreased significantly, AOR = 0.32 CI95% (0.19–0.54). There was no change in smoking or overweight/obese. There was a higher risk of frequent binge drinking and a lower risk of tobacco smoking in all curricula than in nursing students. Medical students practiced sport on a more regular basis, were less overweight/obese, had fewer eating disorders than nursing students.

**Conclusion:**

Our findings demonstrate a stable frequency of classic behaviors as smoking but a worsening of emerging behaviors as binge drinking among healthcare students between 2007 and 2015. Health behaviors differed according to healthcare curricula and nursing students demonstrated higher risks. As health behaviors are positively related to favorable attitudes towards preventive counseling, therefore healthcare students should receive training in preventive counseling and develop healthy lifestyles targeted according to the health curriculum.

## Introduction

The implementation of health-promoting behaviors, such as regular exercise, together with the avoidance of health-risk behaviors such as excessive alcohol consumption, smoking and obesity are essential to reduce the risk of developing serious health problems, such as cancer, cardiovascular disease, and type 2 diabetes [1,2] Available research suggests that the healthy behaviors of physicians influence patients’ attitudes towards preventive counselling and their motivation to make healthy lifestyle choices [3]. Physician-delivered preventive counseling includes advising patients to adopt a healthy diet and do more physical activities [4 5]. Based on the finding that healthy behaviors are positively related to favorable attitudes towards preventive counseling, medical students should receive training in preventive counseling to help promote healthy lifestyles [6, 7]. Healthcare students are the healthcare providers of the future and will not only provide preventive counseling but will also serve as role models and coaches to help enhance their patients’ healthy lifestyle behaviors. Indeed, physicians and medical students who themselves lead healthy lifestyles are more likely to have a positive influence on patients and help them to make healthy choices and lead healthy lives [8, 9, 10]. In addition, other healthcare professionals also provide counseling [11]. The World Health Organization has called on professional healthcare organizations to encourage and support their members to be role models by not using tobacco products and by promoting a tobacco-free culture [12]. Systematic reviews have shown evidence of the efficacy of pharmacists and nurses in delivering alcohol, smoking and weight management interventions in the community [13,14].

Furthermore, healthcare students face multiple stressors, such as academic overload, pressure to succeed, competition with peers and transition to adulthood, as other university students [15, 16]. In addition to this academic context, healthcare students must cope with death and disease during their training in clinical wards [17, 18, 19]. These stressors could lead to adopting risk behaviors as smoking, cannabis use, binge dinking or eating disorders [20]. However, patient relationships and stressors could be different according to the curricula. Accordingly, health behaviours and coping mechanisms could be different according the curricula (medical, nurse, pharmacy and physiotherapist students). Knowledge of risk behaviors among medical students is sparse [21, 22, 23, 24] and there are even fewer studies on nursing, pharmacy and physiotherapy students [25, 26] While much is known about individual lifestyle risk factors among university students, the prevalence and clustering of multiple risk factors has only recently been studied among adolescents [27] and adults [28]. Therefore, it is crucial to assess the changes and specificities in health behaviors among healthcare students in order to develop effective interventions to improve the health and lifestyle behaviors of healthcare professionals. Indeed, healthcare students will be delivering care to people throughout their career and serving as role models and coaches to help enhance their patients’ healthy lifestyle behaviors. Then the hypotheses were that there may be emergent or persistent risk behaviors over a 8-year period among healthcare students and the health behaviour could be different according the field (medical, nurse, pharmacy and physiotherapist students). The aims of the present study were two-fold: first, to assess changes in individual health risk factors and in the composite measure of health risks based on behavioral factors of cancer and cardiovascular disease among healthcare students between 2007 and 2015 [2, 29], and second, to identify specific health behaviors based on the different curriculum of nursing, medical, pharmacy and physiotherapy students.

## Methods

Cross sectional studies were conducted using the same questionnaire in May 2007 and May 2015. The study population comprised healthcare students in Rouen (Normandy, France): nursing, medical, pharmacy and physiotherapy students. All students were invited to fill an anonymous self-administered paper questionnaire. Questionnaire forms were distributed by lecturers to student participants while attending a compulsory course and during an examination session. Questionnaires were distributed to medical and pharmacy students in years 2 through 5, but not in years 1 and 6 due to competititve examinations, and nursing and physiotherapy students in years 1 through 3. Prior to distribution of the questionnaires, lecturers explained the aims of the research study and guaranteed confidentiality of information. The study design was approved by the Commission Nationale de l’Informatique et des Libertés (The French Electronic Data Protection Authority, Declaration number 1353247) and Rouen University Hospital’s Institutional Review Board without mandatory informed consent since the questionnaire was anonymous and self administered-questionnaire.

### Data collection

#### Socio-economic characteristics

The self-questionnaire included socio-economic characteristics: age, gender, grant holder status, student job status, accommodation status (domiciled at parents, rented accommodation, or on campus), and marital status (living with a partner or alone). Data on the curriculum (nurse, medical, pharmacy and physiotherapy) and the academic year of study were also collected.

#### Body mass index

Self-reported height and weight were used to calculate Body Mass Index (BMI) using the standard formula (BMI = weight (kg)/height (m^2^) and classified as: underweight (BMI below 18.5); normal (BMI between 18.5 and 24.9); overweight (BMI between 25.0 and 29.9) and obese (BMI above 30) [30].

#### Practice of sport

Students reported their frequency and duration of practice of sport: strenuous activity (running, jogging, hockey, football, soccer …) and moderate activity (tennis, badmington, swimming.). At least one hour of sport once a week was considered as regular practice.

#### Alcohol use

Binge drinking, defined as consumption of five or more alcoholic drinks (four or more for female students) on any one occasion (of less than two hours in duration), was classified as follows: more than twice a month as frequent, once a month or less as occasional, and total abstinence as never [31].

Consumption of alcohol except binge drinking was classified as follows: on three or more occasions per week as frequent, on one occasion or less per week as occasional, and total abstinence as never. Data on the average number of units of alcohol consumed per occasion were also collected.

#### Tobacco smoking and cannabis use

Tobacco smoking was assed by the cigarette consumption. A current smoker usually smoked at least one cigarette a day. An occasional cannabis consumption was defined as at least one episode in the previous 12 months. A regular cannabis user was defined as using cannabis at least 10 times per month. Tobacco smoking and cannabis use were recorded according to standardized definition [32, 33, 34].

#### Eating disorders

The SCOFF (Sick, Control, One stone, Fat, Food) questionnaire is a screening tool used to identify risk of eating disorders, including anorexia nervosa, bulimia nervosa, and eating disorders not otherwise specified in young adults [35, 36] SCOFF has been translated into French (SCOFF-F) and validated with a internal consistency (alpha Cronbach) is 0.76 [37, 38] It is scored from 0 to 5, according to the number of positive answers. It has been demonstrated to be a highly effective screening instrument, with excellent sensitivity and specificity for the presence of eating disorders (ED) with at least two positive answers. Positive SCOFF indicates there are at least two positive answers to the five yes-or-no questions.

#### Well being measures

Cohen’s 10-item Perceived Stress Scale (PSS) was developed to measure the degree to which individuals appraise their life as stressful and has been widely used in health studies [31, 32]. The French validated version of PSS was used in this study [33]. The quality of the perceived health of health students was assessed with three answers: very good, mostly good, or bad. The lifetime use of psychotropic drugs: anxiolytics, antidepressants and sleeping pills with yes/no answers for each drugs were collected. Lifetime consumption of psychotropic drugs has been defined by at least one positive answer.

#### Composite measure of health risks

Indicators selected for the composite measure were four mofifiable health risks of the HP2020 Objectives [39] and adapted from Adams et al. [28]: smoking, heavy drinking, being overweight-obese and not practising sport on a regular basis. Heavy drinking was defined as on average more than two drinks per day for men or more than one drink per day for women (HP2020 Objective SA-15) [39]. One point was attribued by health risk then the composite measure was scored from 0 to 4. As only 0.8% of students combined 4 risks, they were merged with the group of students who had 3 risks.

### Statistical analysis

Qualitative variables were compared using Pearson’s χ2 test or Fisher’s exact test and quantitative variables were summarized by mean with standard deviation (SD) and compared using the Student’s t-test and ANOVA. A p value below 0.05 was considered to be significant.

Two times trend models were performed to evaluate the independent changes of behavior beetween 2007 and 2015 with the survey year as dependent variable. All possible confounders with a p value below 0.20 in univariate analysis were included in the first logistic regression as socio demographic factors: age, gender, job status, accomodation status, marital status, academic year of study, and behavior: lifetime consumption of psychotropic drugs, regular practice of sport, tobacco smoking, cannabis use, consumption of alcohol, binge drinking. A second model was performed with the composite measure of health risks as independent variables and was adjusted on socio demographic factors: age, gender, grant holder, job status, accomodation status, marital status, academic year of study.

To identify specific health behavior according to the different curriculum of healthcare students (medical, nursing, pharmacy and physiotherapy students), data of the surveys of 2007 and 2015 were pooled. One curriculum model was performed for each health behavior (dependent variables). Then nine curriculum models were performed respectively for body mass index, lifetime use of psychotropic drugs, regular practice of sport, smoking, alcohol consumption, binge drinking, cannabis use, eating disorders and composite measure of health risks. Each model was adjusted on socio demographic data (age, gender, grant holder status, job status, accomodation status, academic year of study), and survey year for potential cohort effect. Colinearity beetween tobacco, alocohol, binge drinking and cannabis use has been assessed. The nursing student group served as the control. Adjusted Odds Ratios (AOR) and their 95% confidence intervals (CI) were calculated. Statistical analysis was conducted using XLSTAT 2016.02.27608.

## Results

In 2007, 1,326 of the overall 1,742 healthcare students participated in the survey (response rate of 76.1%) comprising 485 nursing students (36.6%), 481 medical students (36.2%), 225 pharmacy students (17.0%) and 135 physiotherapy students (10.2%). In 2015, 1,279 of the overall 1,712 healthcare students participated in the survey, (response rate of 74.7%) comprising 253 nursing students (19.8%), 744 medical students (58.2%), 137 pharmacy students (10.7%) and 145 physiotherapy students (11.3%).

### Changes between 2007 and 2015

The baseline characteristics of healthcare students are described in Table 1. The proportion of male students was 26.9% in 2007 and 36.8%. in 2015 (p<0.0001). The mean age of students was 22.0 years (Standard Deviation SD = 3.4) in 2007 and 21.7 years (SD = 2.9) in 2015 (p = 0.04). One quarter of healthcare students had a grant or a student job with no significant difference between 2007 and 2015. Most students lived in rented accommodation and half of them lived with a partner with a significant increase between 2007 and 2015 (respectively p<0.0001 and p<0.0001). The quality of perceived health did not change for healthcare students between 2007 and 2015: respectively, 16.5% and 17.5% for good health, 77.8% and 76.3% for mostly good health and 5.7% and 6.2% for bad health (p = 0.67). (Table 1)

**Table 1:** Baseline characteristics and frequencies of substance use of healthcare students in 2007 and 2015 (N = 2605).

	2007 (n = 1,326)	2015 (n = 1,279)	p
Age (years) mean (SD)	22.0 (3.4)	21.7 (2.9)	0.04
Male gender (%)	26.9	36.8	<0.0001
Student job holder (%)	28.7	26.3	0.16
Student grant holder (%)	26.0	24.3	0.29
Accomodation (%)			<0.0001
At parents	33.1	24.1	
In rented accommodation	61.1	70.8	
On campus	5.8	5.1	
Living with a partner (%)	41.1	52.2	<0.0001
Academic year of study (%)			<0.0001
1	21.6	12.4	
2	29.3	34.9	
3	25.8	26.3	
>3	23.3	30.3	
Body Mass Index (%)			0.73
Underweight	7.8	7.9	
Normal	81.1	81.3	
Overweight	9.1	9.4	
Obese	1.9	1.3	
Stress mean (SD)	14.1 (6.8)	14.0 (6.9)	0.84
Lifetime consumption of psychotropic drugs (%)	30.0	25.0	0.003
Regular practice of sport (%)	63.5	73.7	<0.0001
Duration of sport mean (min/week) (SD)	138.7 (89.7)	158.4 (99.3)	<0.0001
Tobacco smoking (%)	21.3	24.9	0.02
Cigarettes/day mean (SD)	9.5 (5.3)	7.4 (4.6)	<0.0001
Cannabis use (%)			<0.0001
No	80.8	66.9	
Occasional	23.5	30.8	
Regular	4.3	2.3	
Consumption of alcohol (%)			<0.0001
Never	9.3	5.9	
Occasional	74.8	69.7	
Frequent	15.9	24.4	
Binge drinking (%)			<0.0001
Never	39.9	27.3	
Occasional	52.1	58.6	
Frequent	7.9	14.1	
Eating disorder (%)	24.4	23.0	0.41
Composite measure of health risks (%)			0.15
0	42.8	39.7	
1	37.2	37.2	
2	15.2	18.1	
3 and 4	4.8	5.0	

The prevalence of health risk behaviors in 2007 and 2015 are presented in Table 1. There was no significant difference in BMI categories or mean between 2007 and 2015, (21.7 kg/m^2^ (SD = 2.9) in 2007 and 21.8 kg/m^2^ (SD = 2.7) in 2015; p = 0.42) or in eating disorders, respectively 24.4% and 23.0%. The prevalence of regular practice of sport, tobacco smoking, frequent alcohol consumption and binge drinking significantly increased between 2007 and 2015. Half of students had at least one of the health risk behaviors in the composite measure.

Table 2 showed student characteristics and health behaviors between 2007 and 2015 after multivariate analysis (time trend model). There were significant increases in regular practice of sport AOR = 1.33 95%CI (1.1–1.60), occasional and frequent binge drinking, respectively AOR = 1.48 (1.20–1.83) and AOR = 1.73 (1.19–2.51), and a significant decrease in regular cannabis use AOR = 0.32 (0.19–0.54) and use of psychotropic drugs AOR = 0.73 (0.61–0.88). There was an increase in students with any two of the health risk behaviors in the composite measure: AOR = 1.51 95%CI (1.17–1.96) (Table 3).

**Table 2:** Time trend model: changes in health risk behavior between 2007 and 2015 after logistic regression (N = 2605).

	aOR CI95%	p
Lifetime consumpttion of Psychotropic drugs	0.73 (0.61–0.88)	0.001
Regular practice of sport	1.33 (1.10–1.60)	0.002
Tobacco smoking	1.22 (0.98–1.53)	0.08
Cannabis use		
No	1	
Occasional	0.92 (0.74–1.14)	0.43
Regular	0.32 (0.19–0.54)	<0.0001
Consumption of alcohol		
Never	1	
Occasional	0.96 (0.67–1.35)	0.80
Frequent	0.32 (0.87–2.01)	0.19
Binge drinking		
Never	1	
Occasional	1.48 (1.20–1.83)	<0.0001
Frequent	1.73 (1.19–2.51)	0.004

Model adujsted for the variables in the table and socio demographic factors (age, gender, student job, accommodation, living in couple, curriculum, academic year).

**Table 3:** Time trend model: change in composite measure of health risks between 2007 and 2015 after logistic regression (N = 2605).

	aOR CI95%	p
Composite measure of health risks		
0	1	
1	1.12 (0.95–1.34)	0.19
2	1.31 (1.17–1.66)	0.02
3 or 4	1.17 (0.80–1.71	0.43

Model adujsted for socio demographic factors (age, gender, student job, accommodation, living in couple, curriculum, academic year).

### Health behavior based on the curricula of healthcare students

Overall 2,605 healthcare students were included (1,326 in 2007 and 1,279 in 2015), comprising 1225 medical students (47.0%), 738 nursing students (28.3%), 362 pharmacy students (13.9%), and 280 physiotherapy students (10.8%). The baseline characteristics of the healthcare students according to curriculum are described in Table 4.

**Table 4:** Baseline characteristics and frequencies of substance use among nursing, paramedical, pharmacy and medical students (2007 and 2015) (N = 2605).

	Nursing (n = 738)	Physiotherapy (n = 280)	Pharmacy (n = 362)	Medical (n = 1225)	p
Age years Mean (SD)	22.8 (4.98)	21.2 (2.39)	21.7 (1.75)	21.6 (1.78)	<0.001
Male gender (%)	17.5	33.2	34.5	39.0	<0.0001
Student job holder (%)	24.4	20.4	46.8	26.0	<0.0001
Student grant holder (%)	30.5	27.5	19.8	22.6	<0.0001
Accommodation (%)					<0.0001
At parents	34.0	31.8	30.8	23.5	
In rented accommodation	60.9	61.1	62.5	71.7	
On campus	5.1	7.1	6.7	4.8	
Living with partner (%)	45.9	44.0	47.0	47.8	0.56
Academic year of study (%)				5	<0.0001
1	39.3	42.2	0	0	
2	34.1	29.4	30.7	27.3	
3	26.1	27.4	31.3	24.2	
>3	NA	NA	38.0	48.5	
Body Mass Index (%)					0.001
Underweight	8.3	8.6	6.1	7.6	
Normal	77.0	78.7	83.1	84.1	
Overweight	11.7	10.9	8.4	7.5	
Obese	3.0	1.7	8.4	0.7	
Stress Mean (SD)	15.4 (6.8)	14.6 (6.6)	14.7 (7.0)	12.9 (6.9)	0.49
Psychotropic drugs (%)	33.3	30.0	30.6	25.2	0.002
Regular Practice of sport (%)	57.4	66.9	70.3	76.0	<0.0001
Duration of sport mean (min/week) (SD)	138.5 (88.8)	164.5 (103.6)	128.7 (84.9)	155.4 (97.2)	0.001
Tobacco smoking %	30.2	24.4	16.5	20.2	<0.0001
Cannabis use (%)					<0.0001
No	73.2	66.6	77.3	66.0	
Occasional	22.6	29.5	20.2	31.2	
Regular	4.2	3.9	2.5	2.8	
Consumption of alcohol (%)					<0.0001
Never	10.3	6.8	8.6	6.2	
Occasional	76.1	73.5	73.3	69.0	
Frequent	13.6	19.7	18.1	24.8	
Binge drinking (%)					<0.0001
Never	45.2	30.3	34.0	27.7	
Occasional	49.9	57.8	55.0	57.9	
Frequent	4.9	11.9	11.0	14.4	
Eating disorders (%)	30.6	26.0	22.4	18.9	<0.0001
Composite measure of health risks (%)					<0.0001
0	34.9	38.3	43.1	45.5	
1	38.1	38.3	40.6	35.3	
2	19.8	16.6	14.9	15.3	
3 or 4	7.2	6.8	1.4	3.9	

NA: Not Applicable

**Fig 1:**
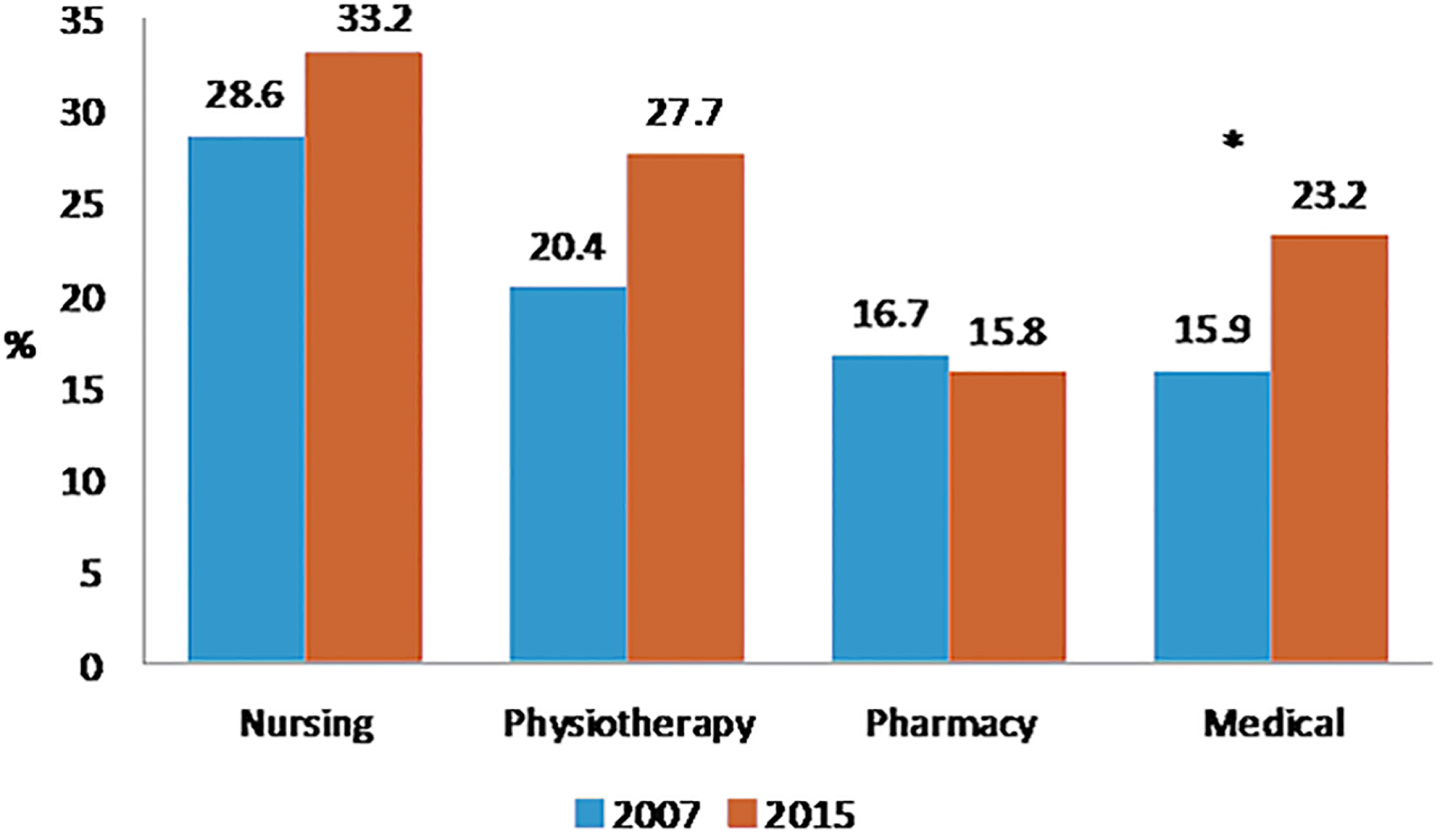
Prevalence of tobacco smoking in 2007 and 2015 according to the curriculum of healthcare students. N = 2605 *:<0.05 **:<0.001.

**Fig 2:**
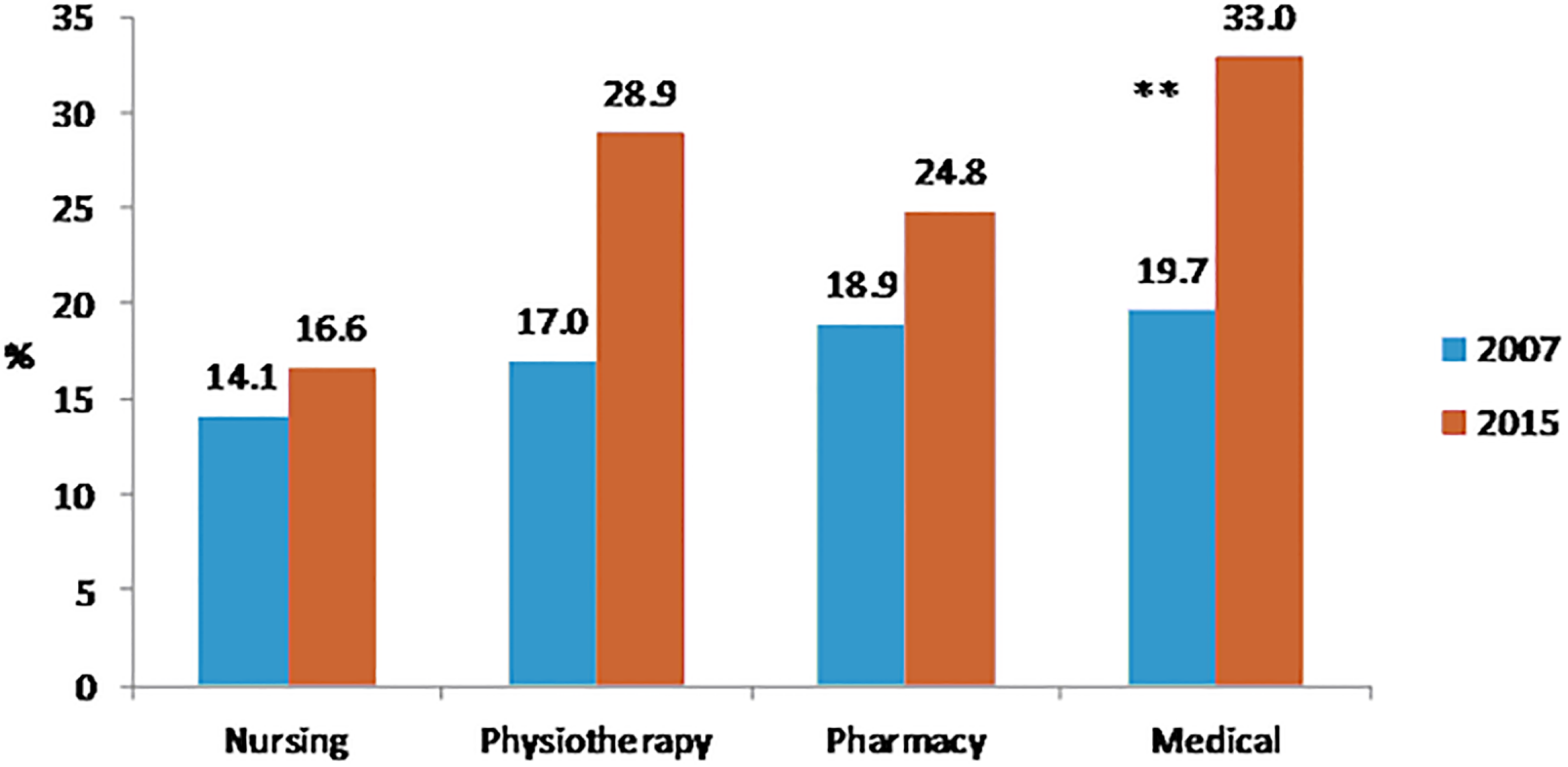
Prevalence of heavy drinking in 2007 and 2015 according to the curriculum of healthcare students. N = 2605 *:<0.05 **:<0.001 Heavy dinking: more than two drinks per day on average for men or more than one drink per day on average for women or frequent binge drinking.

**Fig 3:**
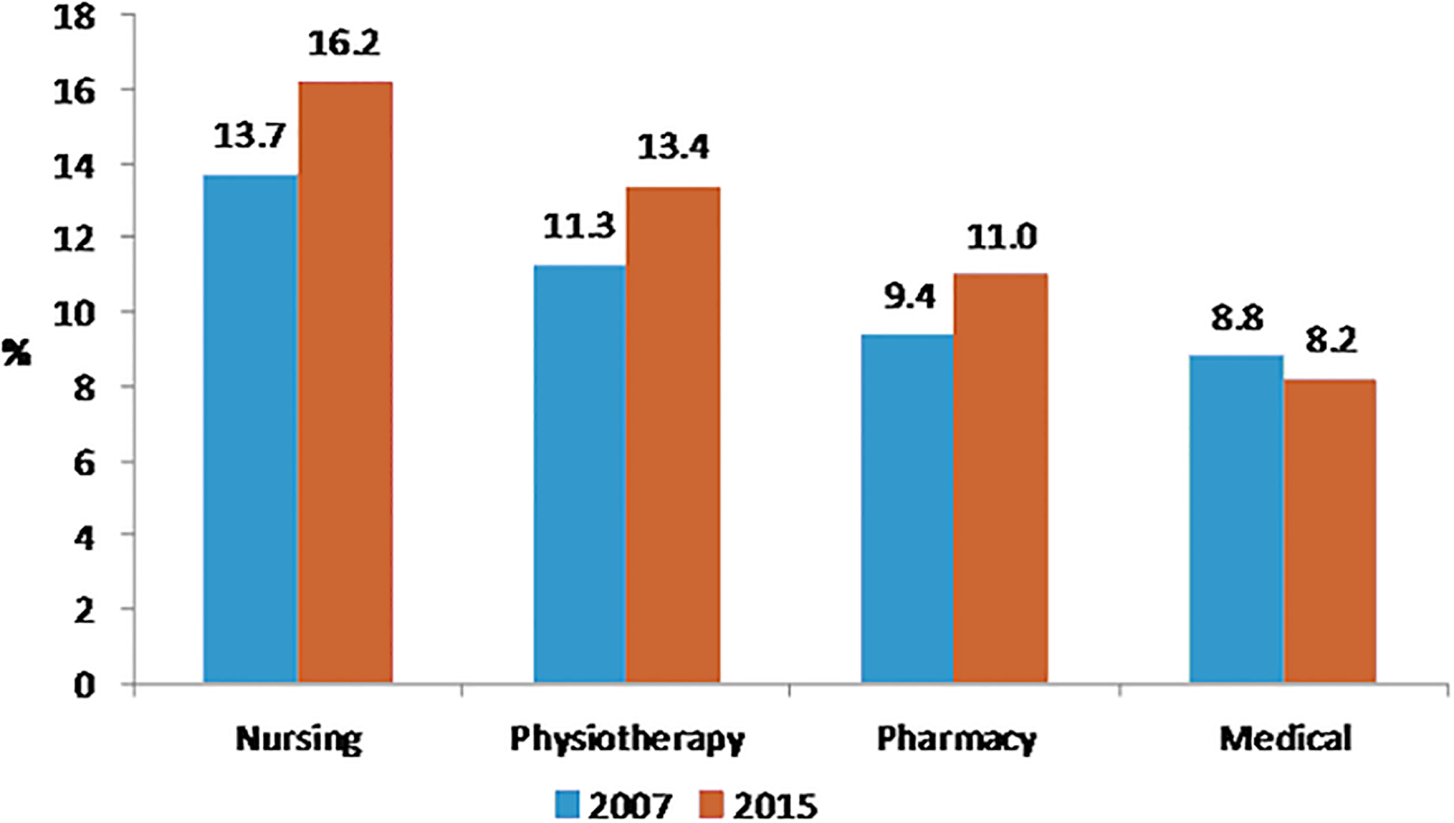
Prevalence of overweight/obesity in 2007 and 2015 according to the curriculum of healthcare students. N = 2605 *:<0.05 **:<0.001.

**Fig 4:**
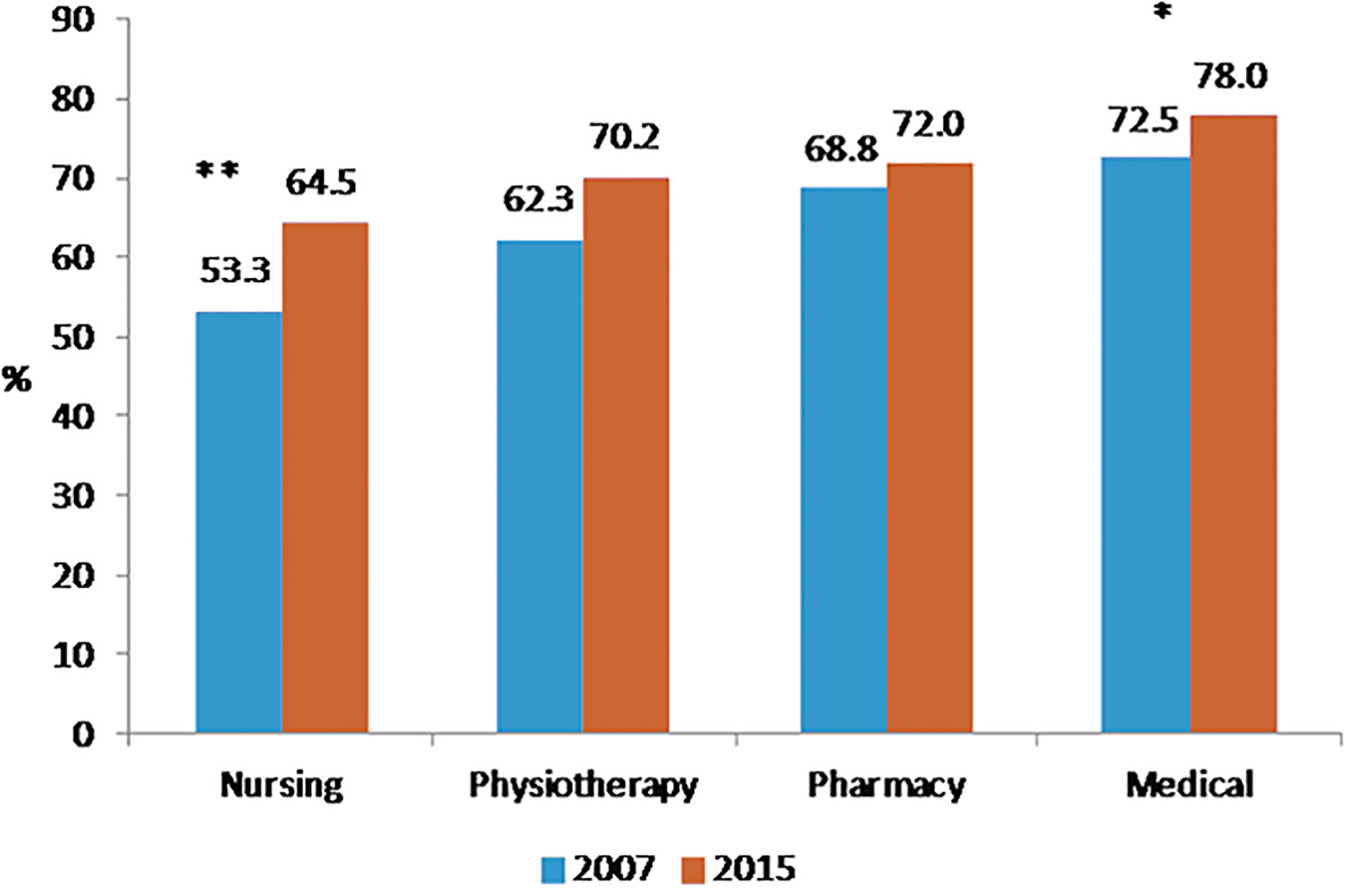
Prevalence of regular practice of sport in 2007 and 2015 according to the curriculum of healthcare students. N = 2605 *:<0.05 **:<0.001.

Perceived health was very good for 21.1% of medical students, 15.4% of paramedic students, 15.1% of pharmacy students and 12.7% of nursing students (p<10^−4^). Figs 1, 2, 3 and 4 showed changes of tobacco smoking, heavy drinking, overweight/obese and regular practice of sport between 2007 and 2015 for each curicula. The prevalence of tobacco smoking was the highest among nursing students (Table 3) and increased significantly among medical students between 2007 and 2015 (Fig 1). The prevalence of heavy drinking was the highest among medical students (28.1%) (14.9% for nursing students, 21.2 for pharmacy students and 23.2% for paramedic students; p<0.0001) and significantly increased among medical and physiotherapy students between 2007 and 2015 (Fig 2). The prevalence of being overweight/obese was the highest among nursing students (Table 4) and remained stable between 2007 and 2015 whatever the disciplines (Fig 3). The prevalence of regular practice of sport was the highest among medical students (Table 4) and increased significantly among medical and nursing students between 2007 and 2015 (Fig 4).

After multivariate analysis, there was an higher risk of frequent binge drinking and a lower risk of tobacco smoking in all curricula except for nursing students. Medical students practiced sport on a more regular basis, were less overweight/obese, had fewer eating disorders and also fewer risk factors in the composite measure of health risks than nursing students (Table 5).

**Table 5:** Curriculum models: specificities in health behaviors according to the curriculum of healthcare students after logistic regression (N = 2605).

	Nursing	Physiotherapy AOR 95%IC	p	Pharmacy AOR 95%IC	p	Medical AOR 95%IC	p
Model 1							
Body Mass Index							
Normal	1	1		1		1	
Underweight	1	1.10 (0.70–1.74)	0.68	0.77 (0.44–1.37	0.38	0.89 (0.56–1.41)	0.62
Overweight and Obese	1	0.90 (0.61–1.33)	0.28	0.79 (0.49–1.29)	0.35	0.64 (0.42–0.97)	0.03
Model 2							
Lifetime of consumption of psychotropic drugs	1	1.13 (0.85–1.50)	0.74	1.14 (0.81–1.60)	0.57	1.08 (0.80–1.44)	0.25
Model 3^a^							
Regular practice of sport	1	1.19 (0.90–1.60)	0.23	1.21 (0.86–1.70)	0.28	1.55 (1.15–2.08)	0.004
Model 4^b^							
Tobacco smoking	1	0.61 (0.44–0.86)	0.005	0.35 (0.23–0.54)	<10^−4^	0.36 (0.25–0.51)	<0.0001
Model 5^c^							
Cannabis use							
No	1	1		1		1	
Occasional	1	0.97 (0.69–1.37)	0.87	0.53 (0.35–0.81)	0.004	0.86 (0.60–1.22)	0.39
Regular	1	0.61 (0.29–1.33)	0.21	0.47 (0.18–1.26)	0.14	0.80 (0.36–1.77)	0.58
Model 6^d^							
Consumption of alcohol		)		)			
Never	1	1		1		1	
Occasional	1	1.31 (0.78–0.20)	0.29	0.77 (0.43–1.37)	0.38	0.75 (0.45–1.23)	0.26
Frequent	1	1.93 (1.03–3.60)	0.04	1.46 (0.72–2.92)	0.29	1.78 (1.00–3.31)	0.05
Model 7^e^	1						
Binge drinking							
Never	1	1		1			
Occasional	1	1.51 (1.10–2.08	0.01	1.43 (0.99–2.09)	0.06	1.23 (0.89–1.70	0.21
Frequent	1	2.26 (1.25–4.08)	0.01	3.23 (1.64–6.35)	0.01	2.88 (1.60–5.17)	<0.0001
Model 8^a^							
Eating disorders	1	0.90 (0.66–1.22)	0.49	0.83 (0.57–1.21)	0.34	0.72 (0.52–0.99)	0.04
Model 9							
Composite measure of health risks				)			
0	1	1		1		1	
1	1	0.93 (0.70–1.25)	0.01	0.85 (0.61–1.18)	0.33	0.66 (0.50–0.89)	0.005
2	1	0.70 (0.49–1.02)	0.07	0.66 (0.43–1.03)	0.07	0.66 (0.46–0.93)	0.02
3 or 4	1	0.82 (0.48–1.41)	0.48	0.15 (0.05–0.42)	<10^−4^	0.41 (0.23–0.73)	0.003

The nine models were adujsted for socio-demographic factors (age, gender, accommodation, job, grant, accommodation, academic year of study) and the survey year.

Added adjusted variables:

^a^ tobacco smoking, cannabis use, consumption of alcohol and binge drinking

^b^ cannabis use, consumption of alcohol and binge drinking

^c^ tobacco smoking, consumption of alcohol and binge drinking

^d^ tobacco smoking, cannabis use and binge drinking

^e^ tobacco smoking, cannabis use and consumption of alcohol

No colinearity beetween alochol, binge drinking, tobacco and cannabis use

## Discussion

### Summary of findings

Our findings demonstrate a stable frequency of classic risk behaviors as smoking but a worsening of emerging behaviors as binge drinking among healthcare students between 2007 and 2015. Our study highlight an increase of regular practive of sport during the period. Health behaviors differed according to healthcare curriculum and nursing students demonstrated higher risks of tobacco smoking, have eating disorders and being overweight/obese than the other healthcare students. Medical students have more regular physical activity than nursing students.

### Interpretation in context of other research

To the best of our knowledge, this is the first study conducted in France with a large sample of healthcare students from the main four health curricula: medicine, nursing, pharmacy and physiotherapy. A second follow-up study was conducted 8 years after the first study, in 2015, allowing us to highlight independent changes in behaviors.

Between 2007 and 2015, there was improvement in two health risk behaviors among healthcare students: regular practice of sport increased and use of regular cannabis decreased. Three in four healthcare students practised sport on a regular basis, which seems higher than for other university curricula in France [40]. Although regular use of canabis decreased howewer it would be interesting to know if healthcare students used other substances for neuroenhancement [41].

Our study shows that the prevalence of tobacco smoking was stable between 2007 and 2015. One in four healthcare students were tobacco smokers, which was similar to other university curricula [32, 40]. We assessed the risk of cancer and cardiovascular disease associated with the use of tobacco. The tobacco product we focused on was the cigarette. In 2007, electronic cigarettes were little used in France and has not been assessed data in our first study. The prevalence of being overweight/obese was 11% and remained stable between 2007 and 2015, similar to other university curricula [42, 43]. Eating disorders also remained stable and involved one in four students. We recently showed that the risk of eating disorders was not higher in healthcare students than in other university students [43].

However, occasional and frequent binge drinking has increased. Indeed, the risk of frequent binge dinking almost doubled over the 8 years between 2007 and 2015. This increase in binge drinking is more wide-spread among university students, than among young people in the general population [44, 45]. However healthcare students did not have especialy high risk of binge drinking [46, 47]. Binge drinking may lead to missed classes, poor academic performance, changes in brain function, lingering cognitive deficit injuries, sexual assaults, overdosing, memory blackouts and death [48, 49] Despite efforts to promote health care, university students continue to engage in high rates of binge drinking [50]. There is growing recognition that post-secondary students including healthcare students should be a target population for health campaigns [51].

Regarding health risks according to curriculum, nursing students were more likely to smoke tobacco than the other healthcare students as already reported [52]. In our study, the prevalence in tobacco smoking among nursing students did no decrease in contrast with the findings reported by Ordas et al. over a 10-year period (30.6% to 19.6%) [53]. These results are significant as a nurse’s personal smoking status influences the attitudes of patients [54] over and above the fact that it is important to avoid smoking in front of their patients. Similar to our findings, other authors have also reported lower levels of tobbaco smoking in physicians than nurses [55]. Healthcare professionals who smoke send an ambiguous message to patients whom they have encouraged to cease smoking [56]. We suggest that healthcare students should be exposed to tobacco control policies and education from the outset of their training [57].

Regarding health risks according to curriculum, nursing students also demonstrated a higher risk of eating disorders, with a prevalence of 30.6%, and a tendency to being overweight/obese (independently of the presence of eating disorders). Shift work for hospital nurses has been recognized as an occupational stressor [58]. Research has indicated that work-related stress and the rotating shift work of nurses induces consumption of foods that are high in sugar, fat, and salt, and a decrease in the consumption of fruits, vegetables, and fiber and of meal frequency [59, 60]. Both stress and shift work are factors that can influence how and what nurses eat and may increase nurses’ risk for weight gain and obesity [61]. Shift work disrupts regular sleep, eating and exercise habits, potentially making it more difficult, to maintain a healthy weight than among other healthcare professionals [62]. Specifically nurses’ working patterns and access to healthy food in the workplace, may also influence a prevalence for overweight and obesity. One of our interesting results was that the medical students practice more regular physical activity than nurses as recently reported by Blake et al [63]. Indeed, in addition to the known cardio vascular protective effect of sport, Cecil et al. found that higher levels of physical activity in medical students were associated with high personal achievement and low emotional exhaustion [64]. In our study; only half of nursing student practised sport on a regular basis. Nurses’ personal levels of physical activity are associated with the frequency of provision of exercise advice. Evidence also suggests that nurses have a role modeling effect insofar as the public is less confident in an overweight nurses’ ability to provide advice on diet and exercise [65].

Regarding the risk of cardio vascular and cancer diseases assessed by the composite measure of health risks (tobacco smoking, heavy drinking, being owerweight/obese or not regular practice of sport), the overall pattern was positive with 45.5% of medical students with no risk compared to 34.9% of nursing students. Medical students also practised sport on a more regular basis than nursing students.

### Limitations

Our studies has some limitations. As study participation was not mandatory the sample might not be representative even though the high response rate limited the bias. The response rates were similar in 2007 and 2015 therefore limiting any potential bias regarding assessment of changes between the two periods. Students might have under reported their own substance use, as this measure was based on self-reporting. Self-reported substance use questionnaires have, however, been shown to be reliable for the substances studied [50, 51]. This study could be lead in other healthcare contexts to have a generalizability of the results.

### Future directions

Healthcare students, especially nursing students, may have unrealistic expectations of their future work and are not adequately prepared to cope with the associated stress, disease of patients and shift work. Assessing the physical health, lifestyle behaviors, and mental health of healthcare students is important to identify health problems and modifiable at-risk behaviors so that early interventions can be implemented to improve outcomes. It is of great importance to identify coping strategies within the framework of career development [66]. Because of additional and potential synergistic effects, multiple-behavior interventions promise to have a greater impact on public health than single-behavior interventions [67]. Programs that develop character and self-awareness enhance the well-being of these future professionals and prepare them to promote the health of their patients [68]. These awareness programs could be part of counseling behavior sessions [69, 70] Early enhancing exposure to learning counseling skills in medical school is likely to be beneficial to the skillset of our future physicians [71].

## Conclusions

Our study highlights the changes in health risk behaviors among the four main healthcare curricula: medicine, nursing, pharmacy, and physiotherapy. Binge drinking seems to concern all curricula and increased over the 8 years between the two studies. The other health risk factors improved or remained stable and do not seem to be more frequent than in other university curricula. Also, we identified nursing students as a target population for prevention and intervention campaigns directed at smoking, eating disorders and being overweight/obese. Healthcare students are future healthcare professionals and as such will be able to provide better care for others when they engage in self-care and promote the highest level of their own well-being [72].

## Supporting information

S1 File Survey questionnaire in French. (DOCX)

S2 File Survey questionnaire in English. (DOCX)

S3 File Database of the two surveys. (XLSX)
