# Supplementary material for: Changes and specificities in health behaviors among healthcare students over an 8-year period
Source: PLoS One. 2018 Mar 22;13(3):e0194188. doi: 10.1371/journal.pone.0194188 (PMC5863977; doi:10.1371/journal.pone.0194188)
Supplement: S1 File — (DOCX) [file pone.0194188.s001.docx]

### Questionnaire « Santé des étudiants »

**Votre âge : ans**

**Votre sexe :**  Homme Femme

**Votre taille (cm) :**

**Votre poids actuel (kg) :**

**Vous êtes :**  Seul En couple

**Vous habitez :**  Chez vos parents Logement personnel Résidence universitaire

**Etudes suivies :**  **Année (1^ère^, etc…) :**

**Avez-vous un travail rémunéré en parallèle de vos études ? :**  OUI NON

**Etes-vous boursier ?**  OUI NON

**TABAC, ALCOOL, CANNABIS**

#### Votre consommation de tabac :

🗆 Vous ne fumez pas

🗆 Vous fumez. **=>** Combien de cigarettes par jour : /__/__/

**Au cours de l'année écoulée, avez-vous bu des boissons alcoolisées ?**

🗆 Jamais (pas une goutte, en aucune occasion) 🗆 2 à 3 fois par semaine

🗆 1 fois par mois ou moins souvent 🗆 Au moins 4 fois par semaine

🗆 2 à 4 fois par mois

**Les jours où vous buvez de l'alcool, combien de verres* buvez-vous ?**

🗆 1 ou 2 🗆 3 ou 4 🗆 5 ou 6 🗆 7 à 9 🗆 10 ou plus

**Combien de fois vous arrive-t-il de boire 6 verres* ou plus au cours d'une même occasion (d'un même repas, d'une même soirée, etc.) ?**

🗆Jamais 🗆Moins d'1 fois/mois 🗆1 fois/mois 🗆1 fois/semaine 🗆Tous les jours ou presque

**Au cours des douze derniers mois, combien de fois avez-vous été ivre en buvant de l'alcool ?**

🗆 Jamais 🗆 1 fois ou 2 🗆 De 3 à 9 fois 🗆 10 fois ou plus

**Avez-vous déjà consommé du cannabis ?** 🗆 OUI 🗆 NON

| Si oui, combien de fois : | Jamais | 1 à 9 | 10 à 30 | > 30 |
| --- | --- | --- | --- | --- |
| **Au cours des 12 derniers mois** | 🗆 | 🗆 | 🗆 | 🗆 |
| **Au cours des 30 derniers jours** | 🗆 | 🗆 | 🗆 | 🗆 |

**VOTRE MORAL**

**Au cours du dernier mois :**

|  | Jamais | Presque jamais | Parfois | Assez souvent | Souvent |
| --- | --- | --- | --- | --- | --- |
| **Avez-vous été dérangé à cause d'un événement inattendu ?** | 🗆 | 🗆 | 🗆 | 🗆 | 🗆 |
| **Vous a-t-il semblé difficile de contrôler les choses importantes de votre vie ?** | 🗆 | 🗆 | 🗆 | 🗆 | 🗆 |
| **Vous êtes vous senti nerveux ou stressé ?** | 🗆 | 🗆 | 🗆 | 🗆 | 🗆 |
| **Vous êtes vous senti confiant à prendre en main vos problèmes personnels?** | 🗆 | 🗆 | 🗆 | 🗆 | 🗆 |
| **Avez-vous senti que les choses allaient comme vous le voulez ?** | 🗆 | 🗆 | 🗆 | 🗆 | 🗆 |
| **Avez-vous pensé que vous ne pouviez pas assumer toutes les choses que vous deviez faire ?** | 🗆 | 🗆 | 🗆 | 🗆 | 🗆 |
| **Avez-vous été capable de maîtriser votre énervement ?** | 🗆 | 🗆 | 🗆 | 🗆 | 🗆 |
| **Avez-vous senti que vous dominiez la situation ?** | 🗆 | 🗆 | 🗆 | 🗆 | 🗆 |
| **Vous êtes vous senti irrité parce que les événements échappaient à votre contrôle ?** | 🗆 | 🗆 | 🗆 | 🗆 | 🗆 |
| **Avez-vous trouvé que les difficultés s'accumulaient à un tel point que vous ne pouviez les contrôler ?** | 🗆 | 🗆 | 🗆 | 🗆 | 🗆 |

**VOTRE ACTIVITE PHYSIQUE**

**Pratiquez-vous une activité sportive?**  OUI NON

**- Si oui, combien de fois par semaine?**

**- Si oui, combien dure une séance?** <30 min 30-60min   >60 min

ALIMENTATION

- **Vous êtes-vous fait vomir parce que vous ne vous sentiez pas bien "l'estomac plein"?**  OUI NON
- **Craignez-vous souvent d'avoir perdu le contrôle des quantités que vous mangez?** OUI NON
- **Avez-vous récemment perdu plus de 6 kg en moins de 3 mois ?**

OUI NON

- **Pensez-vous que vous êtes trop gros(se) alors que les autres vous considèrent comme trop mince ?**  OUI NON
- **Diriez-vous que la nourriture est quelque chose qui occupe une place dominante dans votre vie ?** OUI NON

**VOTRE VISION DES CHOSES**

**De manière générale, pensez-vous que votre état de santé est…**

Très bon Plutôt bon  Mauvais

**Avez-vous déjà pris des tranquillisants, anxiolytiques ?**  OUI NON

**Avez-vous déjà pris des antidépresseurs ?**  OUI NON

**Avez-vous déjà pris des somnifères ?**  OUI NON
