# Supplementary material for: Changes and specificities in health behaviors among healthcare students over an 8-year period
Source: PLoS One. 2018 Mar 22;13(3):e0194188. doi: 10.1371/journal.pone.0194188 (PMC5863977; doi:10.1371/journal.pone.0194188)
Supplement: S2 File — (DOCX) [file pone.0194188.s002.docx]

### Health Campus Study Questionnaire

**Age : years**

**Gender :**  Male Female

**Height (cm) :**

**Weight (kg) :**

**Marital status :**  Living alone Living with a partner

**Living status :**  At parents Rented accommodation On campus

**Curriculum:**  **Years of study:**

**Student job status:**  Yes No

**Grant holder status :**  Yes No

**Tobacco, alcohol and cannabis**

#### Tobacco smoking :

🗆 No smoker

🗆 Smoker. **=>** Number of cigarette/day : /__/__/

**How often do you have a drink containing alcohol?**

🗆 Never

🗆 2 to 3 times a week

🗆 Monthly or less

🗆 2 to 4 times a month

🗆 4 or more times a week

**How many drinks containing alcohol do you have on a typical day when you are drinking?**

🗆 1 to 2 🗆 3 to 4 🗆 5 to 6 🗆 7 to 9 🗆 10 or more

**How often do you have six or more drinks on one occasion?**

🗆Never 🗆Less than monthly 🗆Monthly 🗆Weekly 🗆Daily or almost daily

**Do you ever use cannabis?** 🗆 Yes 🗆 No

| If yes: | Never | 1 to 9 | 10 to 30 | > 30 |
| --- | --- | --- | --- | --- |
| **During the last 12 months** | 🗆 | 🗆 | 🗆 | 🗆 |
| **During the last 30 days** | 🗆 | 🗆 | 🗆 | 🗆 |

**Stress**

**During the last month:**

|  | Never | Almost never | Sometimes | Fairly often | Very often |
| --- | --- | --- | --- | --- | --- |
| 1. In the last month, how often have you been upset because of something that happened unexpectedly? | 🗆 | 🗆 | 🗆 | 🗆 | 🗆 |
| 2. In the last month, how often have you felt that you were unable to control the important things in your life? | 🗆 | 🗆 | 🗆 | 🗆 | 🗆 |
| 3. In the last month, how often have you felt nervous and “stressed”? | 🗆 | 🗆 | 🗆 | 🗆 | 🗆 |
| 4. In the last month, how often have you felt confident about your ability to handle your personal problems? | 🗆 | 🗆 | 🗆 | 🗆 | 🗆 |
| 5. In the last month, how often have you felt that things were going your way? | 🗆 | 🗆 | 🗆 | 🗆 | 🗆 |
| 6. In the last month, how often have you found that you could not cope with all the things that you had to do? | 🗆 | 🗆 | 🗆 | 🗆 | 🗆 |
| 7. In the last month, how often have you been able to control irritations in your life? | 🗆 | 🗆 | 🗆 | 🗆 | 🗆 |
| 8. In the last month, how often have you felt that you were on top of things? | 🗆 | 🗆 | 🗆 | 🗆 | 🗆 |
| 9. In the last month, how often have you been angered because of things that were outside of your control? | 🗆 | 🗆 | 🗆 | 🗆 | 🗆 |
| 10. In the last month, how often have you felt difficulties were piling up so high that you could not overcome them? | 🗆 | 🗆 | 🗆 | 🗆 | 🗆 |

**Sport**

**Practice of sport (except mild activity e.g yoga, easing walking..°**  Yes No

If yes

**- If yes how many times per week?** **1 2 3 3 and more**

**- If yes how long by session ?** <30 min 30-60min   >60 min

Feeding

**Do you make yourself sick because you feel uncomfortably full?**  Yes No

**Do you worry that you have lost control over how much you eat?**  Yes No

**Haveyou recently lost more than 1 stone (6.35 kg) in a 3-month period?**  Yes No

**Do you believe yourself to be fat when others say you are too thin?**  Yes No

**Would you say that food dominates your life?**  Yes No

**VOTRE VISION DES CHOSES**

**How do you perceive your health?**

Very good Mostly good  Bad

**Did you ever use of anxiolytics?**  Yes No

**Did you ever use of antidepressant?**  Yes No

**Did you ever use of sleeping pills?**  Yes No
